# Use and Implications of the Fracture Risk Assessment Tool in Primary Hyperparathyroidism

**DOI:** 10.1001/jamanetworkopen.2026.1549

**Published:** 2026-03-19

**Authors:** Vivek R. Sant, Yaser ElNakieb, Justin F. Rousseau, Yu-Lun Liu, Craig D. Rubin, Naim M. Maalouf

**Affiliations:** 1 Section of Endocrine Surgery, Department of Surgery, UT Southwestern Medical Center, Dallas; 2 Clinical Informatics Center, UT Southwestern Medical Center, Dallas; 3 Department of Neurology, UT Southwestern Medical Center, Dallas; 4 Peter O’Donnell Jr. Brain Institute, UT Southwestern Medical Center, Dallas; 5 Peter O’Donnell Jr. School of Public Health, UT Southwestern Medical Center, Dallas; 6 Division of Geriatric Medicine, Department of Medicine, UT Southwestern Medical Center, Dallas; 7 Department of Internal Medicine and Charles and Jane Pak Center for Mineral Metabolism and Clinical Research, UT Southwestern Medical Center, Dallas

## Abstract

**Importance:**

Although primary hyperparathyroidism (PHPT) increases the risk of osteoporotic fractures, estimation of fracture probability among patients with PHPT remains challenging. Use of the Fracture Risk Assessment Tool (FRAX) among these patients remains poorly characterized, and its utility in identifying patients who may experience fracture risk reduction following parathyroidectomy (PTX) remains unknown.

**Objective:**

To examine the calibration of FRAX in adults with PHPT and to describe the association between estimated probability and differential fracture risk with PTX compared with nonsurgical management.

**Design, Setting, and Participants:**

This retrospective cohort study was performed from January 1, 2000, to January 1, 2024, using the TriNetX electronic health record US dataset. Participants included adults aged 40 to 90 years with diagnosis or biochemistry findings consistent with PHPT, excluding those with missing or out-of-range FRAX parameters.

**Exposures:**

PTX vs nonsurgical management.

**Main Outcomes and Measures:**

Observed major osteoporotic fracture (MOF) and hip fracture. Ten-year FRAX estimations of fracture probability were calculated without bone mineral density (BMD) findings.

**Results:**

Among 59 194 patients with PHPT (mean [SD] age, 65.9 [10.8] years; 44 540 [75.2%] female), 14 783 (25.0%) were treated with PTX. Observed MOF and hip fractures were slightly greater than estimated by FRAX for all deciles of risk, with an MOF calibration y-intercept of 2.0% and slope of 1.17 and hip fracture calibration y-intercept of 1.4% and slope of 1.02. Overall, PTX was associated with a 12% decrease in MOF (hazard ratio [HR], 0.88; 95% CI, 0.77-1.02) and a 13% decrease in hip fracture (HR, 0.87; 95% CI, 0.72-1.07). The MOF score above which PTX was associated with consistently lower MOF hazard was 1.2%, and the corresponding hip score above which PTX was associated with consistently lower hip fracture hazard was 2.7%. Of the cohort not meeting traditional guideline-based surgical criteria, 6522 (25.0%) met this hip score for consistent fracture reduction associated with PTX.

**Conclusions and Relevance:**

In this cohort study of US patients with PHPT, FRAX estimation exhibited acceptable performance. FRAX may effectively stratify fracture risk and inform surgical decision-making even in absence of BMD. A larger subset of patients than previously identified by guidelines may benefit from parathyroidectomy from the standpoint of fracture prevention.

## Key Points

**Question:**

How can the Fracture Risk Assessment Tool (FRAX) be used for patients with primary hyperparathyroidism to guide surgical decision-making?

**Findings:**

In this cohort study of 59 194 patients with primary hyperparathyroidism, observed major osteoporotic fracture and hip fracture events were slightly greater than estimated by FRAX. Compared with nonsurgical management, parathyroidectomy was associated with consistent reduction in major osteoporotic fracture above major osteoporotic fracture scores of 1.2% and hip fracture reduction above hip scores of 2.7%.

**Meaning:**

These findings suggest that FRAX can stratify fracture benefit from parathyroidectomy and identify a larger subset of patients who may benefit from surgical management than considered by current guidelines.

## Teaser

This cohort study assesses the validity of the Fracture Risk Assessment Tool as a means of stratifying parathyroidectomy-associated fracture benefit among patients with primary parahyperthyroidism.

## Introduction

Primary hyperparathyroidism (PHPT) affects nearly 3 million US individuals and causes progressive bone loss resulting in pathologic fractures.^1^ It predominantly affects postmenopausal women, who are already at increased risk of osteoporosis and fracture compared with the general population.^2,3,4,5^ Parathyroidectomy (PTX) is the only durable cure, and although it reduces the risk of fracture and is generally well tolerated, it is not without cost and risk of complication.^6^ Stratification of expected fracture benefit with vs without PTX may thus aid in decision-making regarding PHPT management.

With the advent of automated serum calcium measurements, PHPT in the modern era more commonly presents asymptomatically, but remains underdiagnosed.^7^ The Fifth International Workshop on PHPT^8^ recommends surgical management of PHPT in asymptomatic patients with osteoporosis. However, even certain asymptomatic patients with osteopenia or normal bone mineral density (BMD) have been reported to experience fracture risk reduction with PTX.^4,9,10^ Tools such as the Fracture Risk Assessment Tool (FRAX) have been used to help identify patients in the general population who are at higher fracture risk and may benefit most from pharmacologic therapies for osteoporosis. The Third, Fourth, and Fifth International Workshops on PHPT have identified interest in using FRAX or a similar tool for decision-making in patients with PHPT but note lack of validation of FRAX in this population.^8,11,12^ FRAX has been validated in other specific populations such as patients with cancer, and 10-year fracture and mortality hazards in PHPT without FRAX evaluation have been described previously.^13,14^

To our knowledge, use of FRAX has not yet been validated in patients with PHPT, nor have FRAX intervention thresholds in PHPT been identified. Given the need to better stratify fracture benefit with PTX in PHPT and the potential utility of FRAX (hereinafter *the study tool*) in identifying intervention thresholds, we sought to validate this tool in a large US population of PHPT and identify how it may be used to stratify PTX-associated fracture benefit.

## Methods

This retrospective cohort study using certified deidentified health record data was deemed exempt from review and the need for informed consent by the UT Southwestern Institutional Review Board. We followed the Strengthening the Reporting of Observational Studies in Epidemiology (STROBE) reporting guideline for cohort studies.

### Cohort

A previously described retrospective cohort of adult patients with PHPT was identified from TriNetX, an electronic health record (EHR) dataset representing 100 million patients throughout the US.^4^ Briefly, patients were included by diagnosis code or biochemical diagnosis of PHPT, including patients who underwent PTX and those with nonsurgical management, from January 1, 2000, to January 1, 2024, limited to those with at least 1 year of preinclusion data. Patients were excluded if they had a history of chronic kidney disease (CKD) of stage 4 or greater, dialysis, kidney transplant, or PTX prior to cohort inclusion date. Additionally for this study, patients with missing sex, race and ethnicity, height and/or weight, or parameters out of the range accepted by the study tool (aged <40 or >90 years), were excluded.

### Outcomes

Primary outcomes were first major osteoporotic fracture (MOF), hip fracture, and all fracture events after cohort inclusion, with fracture sites identified via codes from the *International Classification of Diseases, Ninth Revision*, and *International Statistical Classification of Diseases, Tenth Revision*, per prior work (eTable in Supplement 1).^15^ MOF was a composite of the hip, spine, distal forearm, and proximal humerus sites. The Fifth International Workshop guidelines recommend surgical management of PHPT following any nontraumatic fracture, reflecting the expectation that PTX reduces fracture risk beyond hip or MOF sites alone. Accordingly, we also evaluated all nontraumatic fractures, excluding fractures of the skull, hands, and feet.^8^ In patients who experienced a fracture before cohort inclusion, the first fracture after cohort inclusion was defined using a validated claims-based incident fracture algorithm to avoid double-counting prevalent fractures.^16^ Cumulative incidence was assessed, censoring patients at loss to follow-up, and incorporating competing mortality risk.

### Variables

Clinical variables required for study tool probability estimation include age, sex, race and ethnicity (including Asian, Black, Hispanic, and White), height, weight, parental hip fracture history, personal history of fracture, smoking status, glucocorticoid use, rheumatoid arthritis, secondary osteoporosis, and alcohol use. These variables were obtained from the dataset at the time of cohort inclusion, through a previously validated approach for identifying study tool variables from EHR-based datasets.^15^ These variables were entered into the study tool’s calculator (version 1.4.7), and 10-year estimates for MOF and hip fracture were generated for each patient. BMD was not available and was not used for calculation. Parental hip fracture history was unavailable and input as negative. To evaluate the impact of this assumption, a sensitivity analysis was performed in which parental hip fracture history was probabilistically assigned as positive with a prevalence of 10%, based on published estimates in Western populations, across 100 resampled datasets, and study tool estimates and calibration metrics were recalculated.^17^

Additional preinclusion variables relevant to surgical selection were recorded, including presence of CKD diagnosis (stage 3 or higher), kidney stone history, highest serum calcium level, diagnosis of osteoporosis or osteopenia, and prior use of prescription antiosteoporosis medications (including bisphosphonates, denosumab, and teriparatide). Sensitivity analysis excluding patients with prior antiosteoporosis medication use was performed to assess potential confounding by these therapies on association with fracture outcomes.

### Statistical Analysis

Observed probabilities for MOF and hip fracture were estimated at 10 years, incorporating competing mortality risk, using the Aalen-Johansen estimator.^18^ Calibration ratios were calculated by comparing these observed probabilities with estimated probabilities using the study tool, stratifying the cohort by decile and creating a best-fit line by least squares regression to report calibration slopes and intercepts.

Time-dependent area under the receiver operating characteristic curve (AUROC) analysis was performed to assess the performance of 10-year MOF and hip scores both at 10 years and at earlier discrete time points, accounting for censoring and competing risk of mortality and using cumulative sensitivity and dynamic specificity.^19^

To assess the association of PTX with fracture risk reduction and its interaction with the study tool estimate, a Poisson regression was created, estimating the continuous fracture hazard as exp (β_0_ + β_1_ × t + β_2_ × age + β_3_ × FRAX + β_4_ × PTX + β_5_ × FRAX × PTX), with t as time since index date in person-years.^20^ Fracture hazard ratios (HRs) for PTX association (comparing PTX with nonsurgical management) were computed as continuous functions of MOF and hip scores, and for presentation, also reported at discrete intervals. HRs for MOF and hip fracture were modeled as functions of MOF and hip scores, respectively. HRs for all fractures were assessed separately as functions of MOF and hip scores.

To address differences in treatment selection between the PTX and nonsurgical groups, a surgical propensity score was generated using factors expected to contribute to surgical selection, including expected increased fracture risk. These included age, sex, race and ethnicity, inclusion decade, body mass index, alcohol use, smoking status, rheumatoid arthritis, osteopenia, osteoporosis, secondary osteoporosis, fracture history, glucocorticoid use, antiosteoporosis medication use, CKD history, kidney stone history, and highest total serum calcium level prior to cohort inclusion. Our Poisson analysis of PTX and nonsurgical groups was weighted using inverse probability of treatment weighting (IPTW), ensuring covariate balance (standardized mean differences <0.1).

Statistical analyses were conducted with R, version 4.3.1 (R Project for Statistical Computing) and Python, version 3.12.2 (Python Software Foundation). Tests were 2 sided and evaluated at significance threshold *P* < .05. Confidence intervals were calculated at the 95% level. For Poisson analysis, 95% CIs were derived using the delta method based on the covariance matrix from the weighted Poisson regression. For time-dependent AUROC analysis, confidence intervals were computed as asymptotic normal intervals based on influence function variance estimation.

## Results

**Figure 1.**
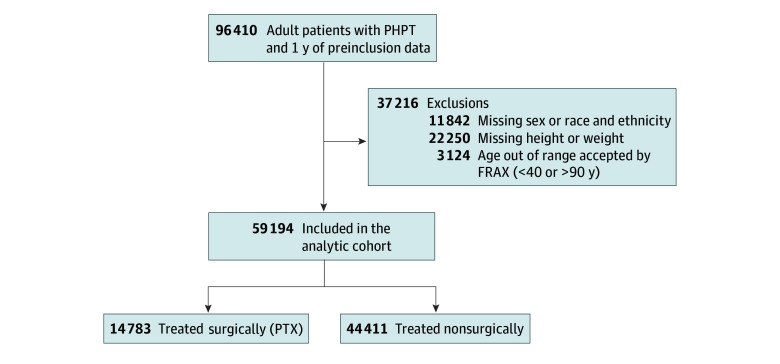
Study Flow Diagram PHPT, primary hyperthyroidism; and PTX, parathyroidectomy.

The final analytic cohort consisted of 59 194 patients with PHPT, with mean (SD) age of 65.9 (10.8) years. A total of 44 540 patients (75.2%) were female and 14 654 (24.8%) were male. In terms of race and ethnicity, 1456 patients (2.5%) were Asian, 13 636 (23.0%) were Black, 2741 (4.6%) were Hispanic, and 41 361 (69.9%) were White. Additional characteristics are described in Table 1. A total of 14 783 patients (25.0%) were treated with PTX, and the remainder were treated nonsurgically (Figure 1). Compared with the nonsurgical group, the PTX group differed slightly, including younger mean age at cohort inclusion, a greater proportion of women, and a lower prevalence of CKD. The weighted cohort had balanced characteristics with a standardized mean difference less than 0.1 (Table 1). Median follow-up was 2.7 (IQR, 0.9-5.6) years. Median time between initial PHPT diagnosis and PTX was 0.3 (IQR, 0.1-0.8) years.

**Table 1.** Characteristics of the PHPT Cohort

Characteristic	Treatment group, No. (%)		SMD	
	PTX (n = 14 783)	Nonsurgical (n = 44 411)	Unweighted	Weighted
Age, mean (SD), y	63.2 (10.0)	66.8 (10.9)	−0.345	−0.033
Sex				
Female	11 577 (78.3)	32 963 (74.2)	−0.041	−0.007
Male	3206 (21.7)	11 448 (25.8)	0.041	0.007
Inclusion year				
2000 to <2010	620 (4.2)	1941 (4.4)	−0.002	0.003
2010 to <2020	7422 (50.2)	21 325 (48.0)	0.022	0.007
≥2020	6741 (45.6)	21 145 (47.6)	−0.020	−0.010
Race and ethnicity				
Asian	453 (3.1)	1003 (2.3)	0.008	0.000
Black	2133 (14.4)	11 503 (25.9)	−0.115	−0.001
Hispanic	737 (5.0)	2004 (4.5)	0.005	−0.002
White	11 460 (77.5)	29 901 (67.3)	0.102	0.003
BMI, mean (SD)	30.4 (7.0)	30.2 (7.3)	0.028	0.014
Highest calcium level				
<11.5 mg/dL	10 811 (73.1)	33 451 (75.3)	−0.022	−0.007
≥11.5 mg/dL	2638 (17.8)	6856 (15.4)	0.024	0.000
Unknown	1334 (9.0)	4104 (9.2)	−0.002	0.007
Comorbidities				
Excessive alcohol use	300 (2.0)	1540 (3.5)	−0.014	−0.001
Current smoking	1556 (10.5)	6082 (13.7)	−0.032	0.003
Rheumatoid arthritis	477 (3.2)	1727 (3.9)	−0.007	0.005
Osteopenia	2541 (17.2)	4472 (10.1)	0.071	−0.002
Osteoporosis	3192 (21.6)	6244 (14.1)	0.075	−0.004
Secondary osteoporosis	2520 (17.0)	9429 (21.2)	−0.042	0.005
Fracture history	1777 (12.0)	6030 (13.6)	−0.016	0.002
CKD stages 3-5	929 (6.3)	7952 (17.9)	−0.116	0.005
Kidney stones	2087 (14.1)	4733 (10.7)	0.035	0.005
Medication use				
Long-term glucocorticoids	10 (0.1)	176 (0.4)	−0.003	0.000
Antiosteoporotic medications	3131 (21.2)	8841 (19.9)	0.013	0.002

Abbreviations: BMI, body mass index (calculated as weight in kilograms divided by height in meters squared); CKD, chronic kidney disease; SMD, standardized mean difference.

SI conversion factor: To convert calcium to mmol/L, multiply by 0.25.

### Fracture Estimation Performance

**Figure 2.**
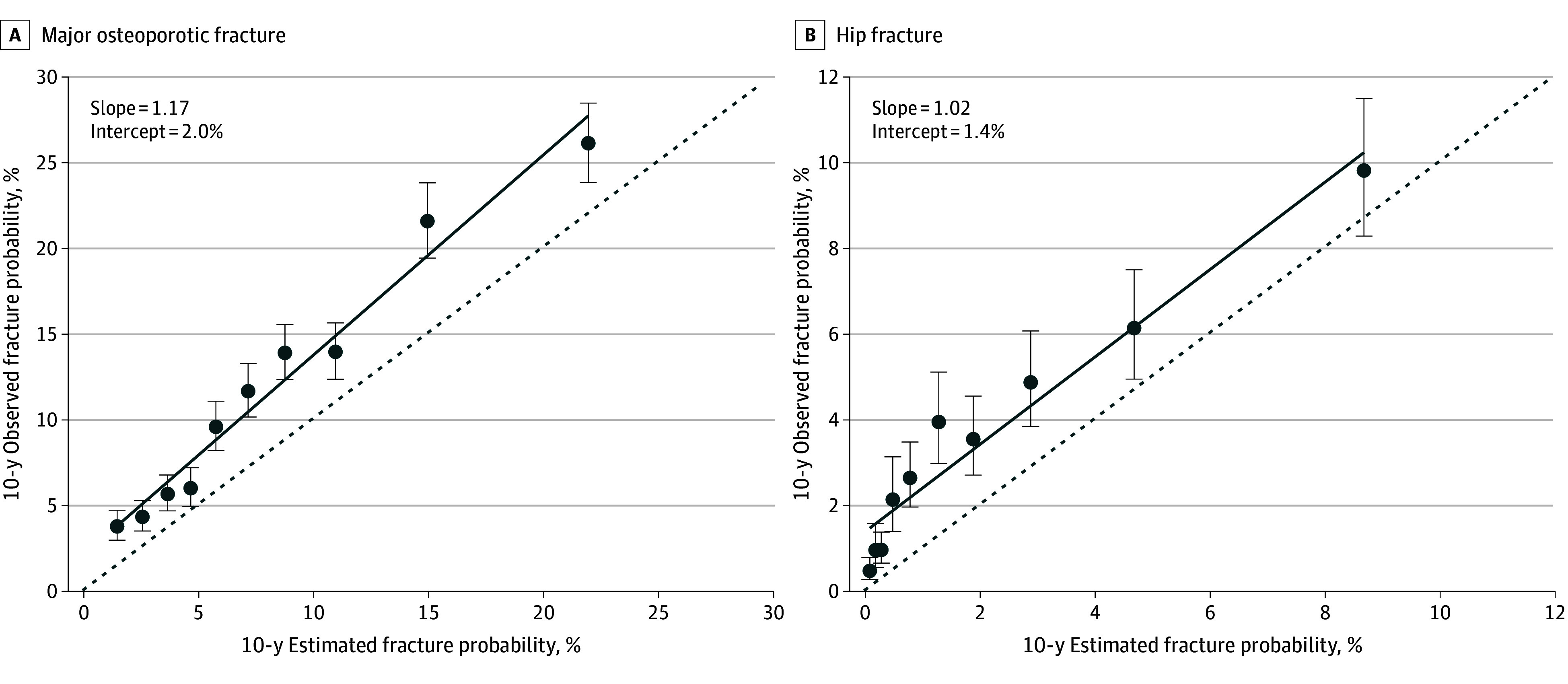
Line Graphs of Calibration Curves of Observed vs FRAX-Estimated 10-Year Fracture Probability for Individuals With Primary Hyperthyroidism The solid diagonal lines indicate calibration best fit; the dashed diagonal lines indicate ideal perfect calibration. Dots indicate observed fracture by decile; error bars indicate 95% CIs.

Calibration curves for the overall cohort are reported in Figure 2 for MOF and hip fracture. Of note, observed MOF was slightly greater than that estimated by the study tool (which underestimated fracture risk for all deciles), with a calibration y-intercept of 2.0% and slope of 1.17. Similarly, hip fracture calibration data points were all above the unity line, with y-intercept of 1.4% and slope of 1.02.

Calibration plots from the sensitivity analysis assuming a 10% prevalence of positive parental hip fracture history are shown in eFigure 1 in Supplement 1. Results from each resampled dataset were stratified by decile, with a linear regression line applied to the mean observed and estimated risks across resamples. For MOF, all points were above the unity line, while for hip fracture, scores were overestimates only at the high end of the scores.

Calibration plots from the sensitivity analysis excluding patients with prior antiosteoporosis medication use are shown in eFigure 2 in Supplement 1. For both MOF and hip fracture, observed events remained slightly greater than estimated by the study tool, with MOF calibration y-intercept of 1.9% and slope of 1.10, and hip fracture calibration y-intercept of 1.0% and slope of 1.14.

Time-dependent AUROC at 10 years for MOF was 0.71 (95% CI, 0.70-0.72) and for hip fracture was 0.75 (95% CI, 0.73-0.77). At earlier times, time-dependent AUROC is shown graphically in eFigure 3 in the Supplement.

### Stratification of Fracture Outcomes by FRAX Score in the IPTW-Cohort

For the weighted cohort, PTX was associated with a 12% decrease in MOF (HR, 0.88; 95% CI, 0.77-1.02) and a 13% decrease in hip fracture (HR, 0.87; 95% CI, 0.72-1.07), suggesting a possible risk reduction, although the confidence intervals included the null. Similarly, PTX was associated with a 7% decrease in all fractures when modeled against MOF score (HR, 0.93; 95% CI, 0.84-1.04) and 7% decrease when modeled against hip score (HR, 0.93; 95% CI, 0.86-1.00).

**Figure 3.**
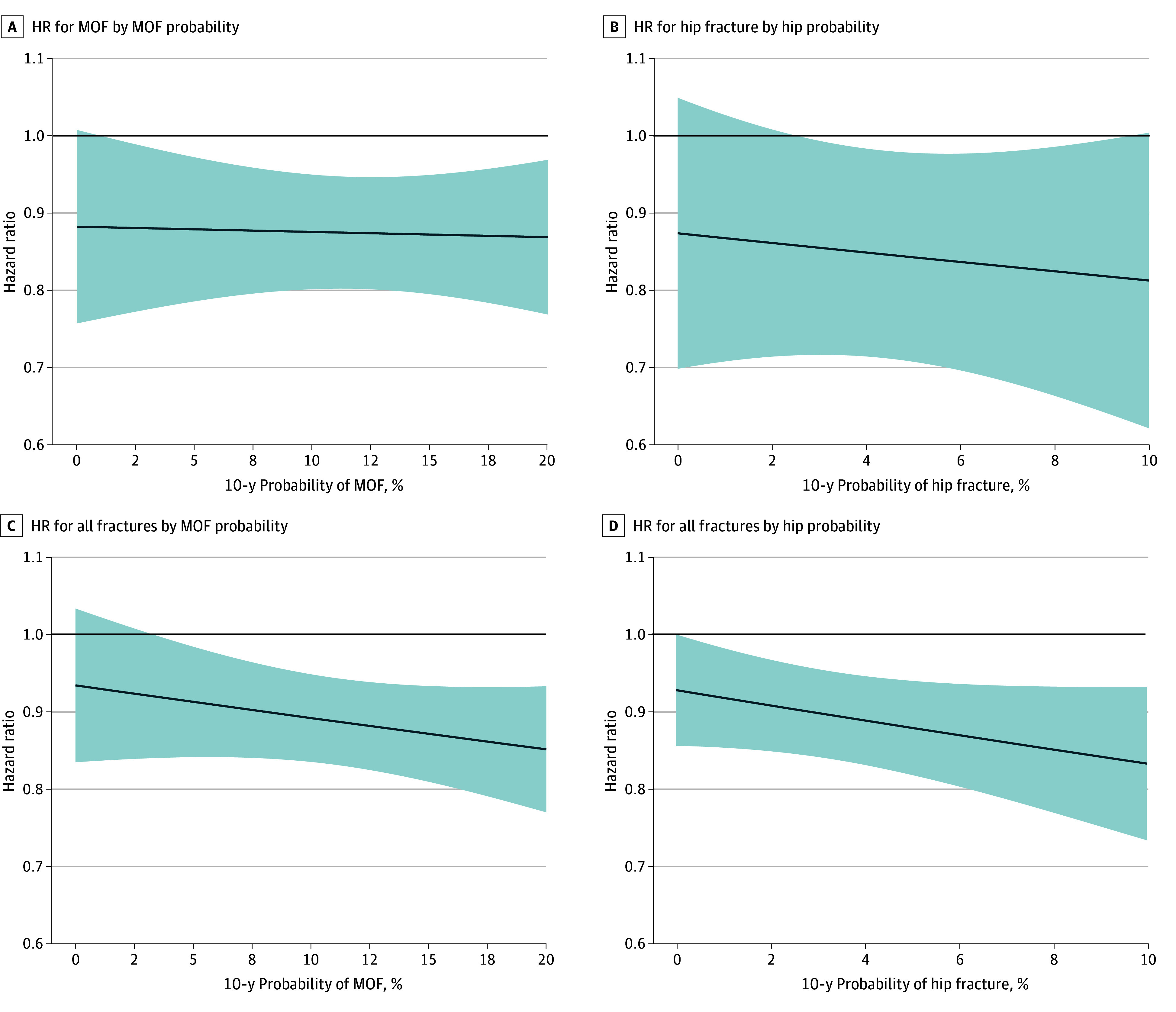
Line Graphs of FRAX Probability and the Association of Parathyroidectomy (PTX) With Fracture The solid horizontal black lines indicate fracture HR of 1.0. The diagonal blue lines indicate fracture HR comparing parathyroidectomy with nonsurgical treatment (HR <1.0 indicates fracture benefit with PTX); the shaded areas indicate 95% CIs. MOF indicates major osteoporotic fracture.

Table 2 summarizes the associations between PTX and fracture outcomes at discrete scores for MOF, hip, and all fractures, with corresponding continuous estimates shown in Figure 3. PTX was consistently associated with reduced fracture risk above scores of 1.2% for MOF, 2.7% for hip fracture, and 3.2% for all fractures based on MOF score. PTX was associated with reduced risk of all fractures across all hip scores. Interaction terms between score and PTX were not significant (MOF-MOF HR, 0.93 [95% CI, 0.35-2.46; *P* = .83]; hip-hip HR, 0.49 [95% CI, 0.025-9.49; *P* = .63]; MOF–all fracture HR, 0.63 [95% CI, 0.20-1.38; *P* = .25]; hip–all fracture HR, 0.34 [95% CI, 0.08-1.46; *P* = .15]). Nonetheless, both Table 2 and Figure 3 demonstrate that the HR for the association of PTX with fracture decreased progressively with increasing score. A more pronounced fracture reduction at higher scores was seen in hip fracture, compared with that seen in MOF, and the reduction was most pronounced for all fractures.

**Table 2.** Fracture HRs Comparing Treatments (PTX vs Nonsurgical) at Different Values of FRAX 10-Year Probability of MOF and Hip Fracture^a^

Fracture site	Association between PTX and fracture outcomes by discrete FRAX score									
	10th Percentile		25th Percentile		50th Percentile		75th Percentile		90th Percentile	
	Score, %	HR (95% CI)	Score, %	HR (95% CI)	Score, %	HR (95% CI)	Score, %	HR (95% CI)	Score, %	HR (95% CI)
MOF	2.0	0.88 (0.77-0.99)	3.6	0.88 (0.79-0.97)	6.4	0.88 (0.80-0.95)	11.0	0.87 (0.80-0.95)	17.0	0.87 (0.77-0.97)
Hip fracture	0.1	0.87 (0.70-1.04)	0.3	0.87 (0.71-1.03)	1.0	0.86 (0.72-1.00)	2.9	0.84 (0.71-0.98)	6.1	0.83 (0.67-0.98)
All fractures										
MOF	2.0	0.93 (0.84-1.01)	3.6	0.91 (0.84-0.98)	6.4	0.89 (0.83-0.95)	11.0	0.87 (0.81-0.93)	17.0	0.85 (0.77-0.93)
Hip fracture	0.1	0.92 (0.85-0.99)	0.3	0.92 (0.85-0.98)	1.0	0.90 (0.85-0.96)	2.9	0.88 (0.82-0.94)	6.1	0.86 (0.78-0.93)

Abbreviations: HR, hazard ratio; MOF, major osteoporotic fracture; PTX, parathyroidectomy.

^a^ Estimates are derived from a continuous model and evaluated at prespecified scores. HRs compare PTX and nonsurgical treatment.

### Proportion of Cohort Expected to Experience PTX-Related Fracture Benefit

The Fifth International Workshop guidelines recommend PTX in patients with PHPT who are asymptomatic but are younger than 50 years, exhibit osteoporosis on dual x-ray absorptiometry scan, nephrolithiasis, or CKD of stage 3 or higher.^8^ Of our overall cohort, 26 136 (44.2%) met none of the above criteria. Of those not meeting any of the above surgical criteria, 6522 of 26 136 (25.0%) had a hip score greater than 2.7% and 25 926 of 26 136 (99.2%) had an MOF score greater than 1.2%, for consistent hip fracture and MOF reduction associated with PTX, respectively. For consistent reduction in all fractures, 26 136 (100%) had a hip score greater than 0 and 21 927 (83.9%) had an MOF score greater than 3.2%.

## Discussion

In this large US cohort of PHPT, the study tool demonstrated acceptable performance estimating MOF and hip fracture, as evidenced by consistent calibration with observed fractures. Fracture benefit with PTX is more apparent above specific scores, and hip fracture reduction may be more pronounced with increasing baseline scores. Collectively, these findings suggest that the study tool may have utility in surgical decision-making for the management of PHPT by identifying which patients may benefit most from PTX. Our findings also suggest that a larger proportion of patients may experience fracture reduction from PTX than those identified by current guideline recommendations.

Although the study tool did not explicitly account for PHPT, its calibration and AUROC for both MOF and hip fracture in our population with PHPT appeared reasonable. Development of the study tool’s calculator involved calibration across broad diverse populations, and it has performed well in other subpopulations with higher anticipated baseline fracture risk, such as patients with cancer.^13,21^ Our findings in patients with PHPT align with this prior work and suggest a strong impact of the other clinical risk factors that are accounted for by the study tool. The consistent slight underestimation of MOF and hip fracture may be due to the additional impact of PHPT on fracture risk that is not accounted for by the tool. This is in line with findings by Kanis et al^14^ that patients with PHPT had higher mean 10-year fracture probabilities than age-matched population controls, although they did not explicitly report estimates for either population. Our demonstrated calibration with simulated parental hip fracture history was relatively stable for MOF, with consistent slight underestimates at all MOF deciles, while at higher hip deciles, hip fracture was somewhat overestimated. Excluding patients with prior antiosteoporosis medication use yielded minimal change in MOF and hip fracture calibration. Finally, the consistent time-dependent AUROC performance at several earlier times suggests utility of the study tool in estimating fracture probability among patients with PHPT even prior to 10 years.

Our work has important clinical implications, as PHPT is a common condition that impacts more than 3 million adults in the US and is increasingly diagnosed in an asymptomatic stage. We note that a large proportion of patients with PHPT may benefit from PTX by way of fracture risk reduction, as benefit is apparent at even low scores. In fact, 25.0% of the patients in our cohort not previously meeting guideline-based criteria for surgery would likely benefit from PTX with respect to hip fracture reduction, 99.2% may benefit by MOF reduction, and nearly all patients may benefit by all fracture reduction. Benefit at all fracture sites was slightly more modest than at hip or MOF sites but appeared more consistent. If confirmed in other cohorts, our findings may allow clinicians to expand consideration of fracture benefit in patients with PHPT beyond the traditional recommendation of osteoporosis on BMD testing. This is concordant with a growing evidence base that fracture risk among patients with PHPT may depend on many variables beyond BMD alone and that fracture risk reduction with PTX may be experienced by a larger portion of patients.^9,14,22^ While the study tool was not created with PHPT or PTX decision-making in mind, intervention thresholds have been successfully used in the general population to identify which patients may benefit from BMD testing, or which patients may benefit from pharmacological intervention to impact fracture risk.^20,23^ Analogous use of the study tool in PHPT may thus have similar value.

In our study, hip fracture and all fracture reduction with PTX appeared greater at higher hip scores, although this interaction did not meet statistical significance. Prior work has noted greater fracture risk reduction after PTX in those with more progressive BMD loss.^4,9,10^ Similarly, antiresorptive medications in the general population have shown progressive fracture benefit at higher scores.^20^ Given these findings, we had expected to see greater benefit at higher scores in patients with PHPT, which is somewhat apparent for all fractures and hip fracture, although less clear in MOF. This may be explained by the findings of Kanis et al,^14^ who note that among patients with PHPT, non–BMD-related fracture risk factors also portend a higher competing mortality hazard, which they found mitigated the fracture benefit when considering overall fracture probability. Also, while we adjusted for baseline differences in antiosteoporosis medication use with weighting, the retrospective study design does not account for nonrandom initiation of antiosteoporosis medication therapy among higher-risk patients during follow-up, potentially attenuating the observed benefit of PTX. The attenuated HR decline at higher MOF scores compared with hip fracture scores may be due to differential benefit among the constituent MOF sites compared with the hip alone. Hip primarily represents cortical bone, as does forearm, while spine and proximal humerus have a greater representation of trabecular bone. Trabecular bone was originally suggested to be adversely affected to a lesser degree or at a later stage by PHPT, although more recent studies call into question this belief.^24,25^ Regardless of etiology, the constituent sites for MOF appear to experience differential benefit with PTX, in line with our findings.^26^

### Limitations

This study has some limitations. Our study design was retrospective, with likely nonrandom selection of patients to undergo PTX compared with nonsurgical management. As with all observational studies of treatment decisions, residual confounding by indication cannot be fully excluded, even after adjustment using a surgical propensity score and IPTW; accordingly, observed associations should be interpreted cautiously. Parental hip fracture history was not available in our cohort, which is a relevant input variable for study tool estimation. However, sensitivity analysis simulating expected rates of parental hip fracture history did not show a major change in our findings. As with any EHR-based retrospective study, we are limited by potential coding inaccuracies and patients receiving portions of their care outside institutions captured in this dataset. Our decision to start t = 0 for the PTX group at date of PTX may introduce a survivor bias; however, given the short interval between PHPT diagnosis and PTX, and similar prevalence of fracture history before t = 0 between the PTX and nonsurgical groups, we expect any bias introduced by this decision to be modest. Finally, we highlight our use of the study tool without BMD: fracture estimation is affected by BMD when provided. However, the study tool may be used without BMD input, and clinicians do not always have ready access to a patient’s BMD. Our approach and findings provide support to a clinician who wishes to complement surgical decision-making in PHPT with non-BMD clinical risk factors.

## Conclusions

The findings of this cohort study suggest that FRAX may be a valid means of stratifying which patients with PHPT may experience fracture benefit with PTX, even in the absence of BMD. Applying FRAX in this manner can identify additional patients who may derive benefit from PTX beyond those captured by current guideline criteria and warrants external and prospective validation.

## Notes

Supplement 1. **eTable.** Diagnosis Codes for Fracture Site Identification **eFigure 1.** Sensitivity Analysis of Parental Hip Fracture History Based on 100 Simulated Datasets With Positive Parental Hip Fracture History Set at 10% Prevalence **eFigure 2.** Sensitivity Analysis Including Only Patients Without History of Antiosteoporosis Medication Use **eFigure 3.** Time-Dependent Area Under the Receiver Operating Characteristic Curve

Supplement 2.
Data Sharing Statement
