# Supplementary material for: Use and Implications of the Fracture Risk Assessment Tool in Primary Hyperparathyroidism
Source: JAMA Netw Open. 2026 Mar 19;9(3):e261549. doi: 10.1001/jamanetworkopen.2026.1549 (PMC13003369; doi:10.1001/jamanetworkopen.2026.1549)
Supplement: Supplement 1. — eTable. Diagnosis Codes for Fracture Site Identification eFigure 1. Sensitivity Analysis of Parental Hip Fracture History Based on 100 Simulated Datasets With Positive Parental Hip Fracture History Set at 10% Prevalence eFigure 2. Sensitivity Analysis Including Only Patients Without History of Antiosteoporosis Medication Use eFigure 3. Time-Dependent Area Under the Receiver Operating Characteristic Curve [file jamanetwopen-e261549-s001.pdf]

Supplementary Online Content

Sant VR, ElNakieb Y, Rousseau JF, Liu YL, Rubin CD, Maalouf NM. Use and implications of the Fracture Risk Assessment Tool in primary hyperparathyroidism. *JAMA Netw Open.* 2026;9(3):e261549. doi:10.1001/jamanetworkopen.2026.1549

eTable. Diagnosis Codes for Fracture Site Identification
eFigure 1. Sensitivity Analysis of Parental Hip Fracture History Based on 100 Simulated Datasets With Positive Parental Hip Fracture History Set at 10% Prevalence
eFigure 2. Sensitivity Analysis Including Only Patients Without History of Antiosteoporosis Medication Use
eFigure 3. Time-Dependent Area Under the Receiver Operating Characteristic Curve

This supplementary material has been provided by the authors to give readers additional information about their work.

© 2026 Sant VR et al. *JAMA Network Open*.

eTable. Diagnosis Codes for Fracture Site Identification

Fracture Site	ICD-9	ICD-10
Hip	820, 733.14, 733.96	S72.0, S72.1, S72.21, S72.22, S72.23, S72.24, S72.25, S72.26, S79.0, M84.459, M84.659
Spine	805, 806, 733.13	S12, S22.0, S22.1, S32.0, S32.1, S32.2, S34.1, M48.4, M48.5, M80.08, M80.88
Proximal humerus	812.0, 812.1, 812.2, 812.3, 733.11	S42.2, S42.3, S49.0, M80.02, M80.82, M84.42, M84.62
Distal forearm	813.4, 813.5, 733.12	S52.5, S52.6, S59.0, S59.2, M80.03, M80.83
Shoulder	810, 811	S42.0, S42.1, S42.9, M80.01, M80.81, M84.31, M84.41, M84.61
Distal humerus	812.4, 812.5	S42.4, S49.1, M84.32
Proximal forearm	813.0, 813.1, 813.2, 813.3, 813.8, 813.9	S52.0, S52.1, S52.2, S52.3, S52.9, S59.1, M84.33, M84.43, M84.63
Ribs & sternum	807.0, 807.1, 807.4, 807.2, 807.3	S22.3, S22.4, S22.5, S22.9, S22.2
Pelvis	808, 733.98	S32.3, S32.4, S32.5, S32.6, S32.8, S32.9, M80.0, M80.8, M84.35, M84.650
Femur (non-hip)	821, 733.15, 733.97	S72.3, S72.4, S72.8, S72.9, S79.1, M80.05, M80.85, M84.45, M84.651, M84.652, M84.653, M84.75
Lower leg	823, 733.16, 733.93	S82, S89.0, S89.1, S89.2, S89.3, M80.06, M80.86, M84.36, M84.46, M84.66

© 2026 Sant VR et al. *JAMA Network Open*.

eFigure 1. Sensitivity Analysis of Parental Hip Fracture History Based on 100 Simulated Datasets With Positive Parental Hip Fracture History Set at 10% Prevalence

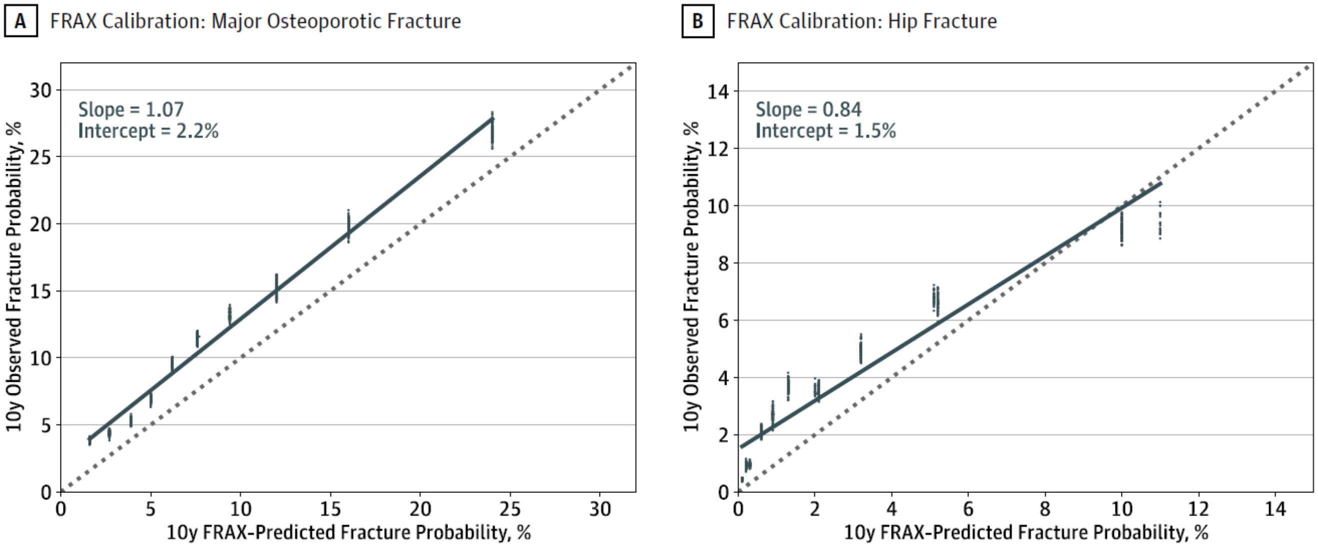

© 2026 Sant VR et al. *JAMA Network Open*.

eFigure 2. Sensitivity Analysis Including Only Patients Without History of Antiosteoporosis Medication Use

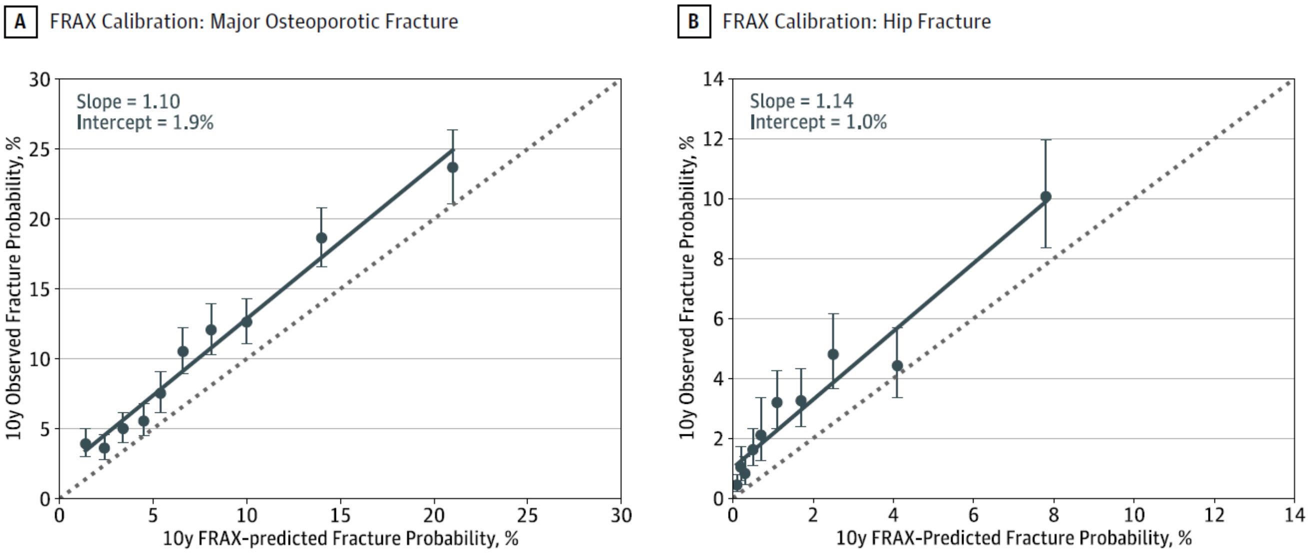

© 2026 Sant VR et al. *JAMA Network Open*.

eFigure 3. Time-Dependent Area Under the Receiver Operating Characteristic Curve

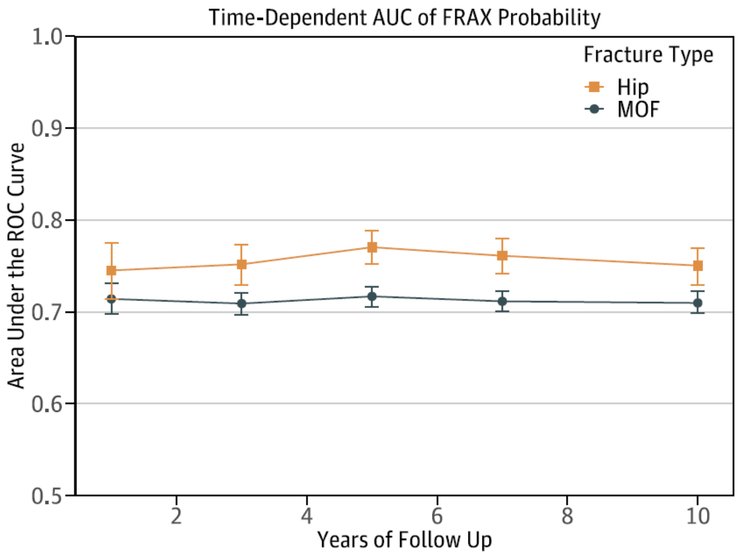

© 2026 Sant VR et al. *JAMA Network Open*.
