# Supplementary material for: Use and Implications of the Fracture Risk Assessment Tool in Primary Hyperparathyroidism
Source: JAMA Netw Open. 2026 Mar 19;9(3):e261549. doi: 10.1001/jamanetworkopen.2026.1549 (PMC13003369; doi:10.1001/jamanetworkopen.2026.1549)
Supplement: Supplement 2. — Data Sharing Statement [file jamanetwopen-e261549-s002.pdf]

Data Sharing Statement

Sant. Use and Implications of the Fracture Risk Assessment Tool in Primary Hyperparathyroidism. *JAMA Netw Open*. Published March 19, 2026. doi:10.1001/jamanetworkopen.2026.1549

Data
Data available: No
